# Effect of haemoglobin concentration on the clinical outcomes in patients with acute myocardial infarction and the factors related to haemoglobin

**DOI:** 10.1186/1756-0500-4-142

**Published:** 2011-05-22

**Authors:** Quan-Zhou Feng, Yu-Sheng Zhao, Yu-Feng Li

**Affiliations:** 1 Institute of Geriatric Cardiology, Chinese PLA General Hospital, Fuxing Road 28, Beijing, 100853 China

## Abstract

**Background:**

The impact of haemoglobin concentrations on clinical outcomes is still a controversial issue. To determine the association between haemoglobin concentrations on admission and clinical outcomes and the related factors, this study was performed in a Chinese hospital.

**Findings:**

We conducted a retrospective study on 1394 Chinese patients with acute myocardial infarction. Patients were categorized according to the haemoglobin concentration on admission, and data were evaluated to determine whether there was an association between the haemoglobin concentrations on admission and 30-day in-hospital MACEs (major cardiovascular events). Patients with hemoglobin values between 141 and 150 g/L were used as the reference, the MACEs increased as hemoglobin concentrations fell below 140 g/L or rose > 150 g/L, with an adjusted OR (odds ratio) of 5.96[95% CI (confidence interval) 2.00 to 17.68, p = 0.0013], 4.39(1.37 to 14.08, p = 0.0128), 3.99(1.46 to 10.92, p = 0.0071), 3.19(1.27 to 8.05, p = 0.0139), 2.37(0.94 to 6.01, p = 0.0687), 2.11(0.66 to 6.74, p = 0.2065), 2.01(0.60 to 6.68, p = 0.2559) in patients with haemoglobin concentrations <100 g/L, 101-110 g/L, 111-120 g/L, 121-130 g/L, 131-140 g/L, 151-160 g/L, and >160 g/L respectively. Partial correlation analysis showed that age, albumin and creatinine were significantly associated with hemoglobin concentration.

**Conclusions:**

Our results demonstrated that haemoglobin concentration affected MACEs in patients with acute myocardial infarction, and that haemoglobin concentration was associated with age, albumin and creatinine.

## Background

Myocardial infarction results from the imbalance of the oxygen supply and demand of the jeopardized myocardium. Anemia has been reported to be present in 15% of patients presenting with acute myocardial infarction (AMI) [1] and in 43% of elderly patients with AMI [2]. Anemia has the potential to worsen the myocardial ischemic insult in AMI, both by decreasing the oxygen content of the blood supplied to the jeopardized myocardium [3] and by increasing myocardial oxygen demand through necessitating a higher cardiac output to maintain adequate systemic oxygen delivery [4]. Therefore, it is hypothesized that haemoglobin (Hb) concentrations on admission affect the prognosis of patients with myocardial infarction.

In animal models, higher hemoglobin concentrations prevent ischemia in the setting of significant coronary artery stenoses [5,6]. Transfusion of anemic animals up to 100 g/L Hb with fresh blood reduces infarct size and improves cardiac function, but, transfusion to 120 g/L Hb did not demonstrate any additional benefit and was associated with larger infarcts [7]. In human studies, anemia has been shown to be an independent risk factor for adverse cardiovascular outcomes in community cohorts[8], in patients with heart failure[9,10], in patients undergoing percutaneous coronary intervention (PCI) [11] and in acute coronary syndrome[12-16]. In the cohort study including nearly 40 000 patients with acute coronary syndrome (ACS), a reverse J-shaped relationship between baseline hemoglobin values and major adverse cardiovascular events was reported [17]. The development of anemia during hospitalization for AMI was associated with an increased long-term mortality [18]. But anemia in some studies was not found to have a higher mortality [15], and blood transfusion in the setting of acute coronary syndromes is associated with higher mortality [19,20]. Furthermore, the risk factors relating to hemoglobin concentrations were not investigated in the studies. Therefore, we examined the relationship between baseline hemoglobin concentration and cardiovascular clinical outcomes in Chinese patients with AMI and investigated the risk factors relating to hemoglobin concentrations.

## Methods

### Study patients

The study cohort eligible for these analyses consisted of 1394 patients with AMI hospitalized in Chinese People's Liberation Army General Hospital between Jan 1998 and Aug 2009. Patients with active cancer (having cancer and hemoglobin concentration < 141 g/L on admission), significant liver (aspartate aminotransferase or alanine aminotransferase >40 × 2 U/L and serum albumin <40 g/L) or renal disease (a creatinine > 177 μmol/L), active or recent (past three months) internal bleeding, known bleeding diathesis were excluded from these study.

Written informed consents were obtained from the patients for publication of this short report. This study protocol has been approved by the committee on human research of Chinese People's Liberation Army General Hospital.

### Data collection and group comparisons

Baseline data were collected retrospectively by use of a standardized form including past history, smoking history, family history of coronary heart disease (CHD), hypertension history, location of myocardial infarction, left ventricular ejection fraction (LVEF) by echocardiography in one week after admission, heart rate (mean value of heart rates within 24 hours after arrival), blood pressure, laboratory data on admission (within 24 hours after arrival at the hospital) and medication.

According to the hemoglobin concentration values on admission (the first measurement taken within 24 hours after arrival at the hospital), the patients were divided into eight categories: Group <100 g/L; Group 101-110 g/L; Group 111-120 g/L; Group 121-130 g/L; Group 131-140 g/L; Group 141-150 g/L; Group 151-160 g/L; Group >160 g/L for the purpose of evaluating the associations among the hemoglobin on admission, various characteristics of the patient, and major cardiovascular events (MACEs).

### Major cardiovascular events

Major cardiovascular events included cardiac mortality within 30 days after admission and in-hospital complications (the development of cardiogenic shock, the occurrence of congestive heart failure, postinfarction angina, and ventricular tachycardia/fibrillation).

### Definitions

AMI is defined as typical rise and fall creatine kinase-MB (CK-MB) of biochemical markers of myocardial necrosis with at least one of the following: a) ischemic symptoms; b) development of pathologic Q waves on the ECG; c) Electrocardiogram (ECG) changes indicative of ischemia (ST segment elevation or depression); or d) coronary artery intervention (e.g., coronary angioplasty)[21].

Cardiogenic shock is defined as hypotension (a systolic blood pressure of less than 90 mmHg for at least 30 minutes or the need for supportive measures to maintain a systolic blood pressure of greater than or equal to 90 mmHg), end-organ hypoperfusion (cool extremities or a urine output of less than 30 ml/h, and a heart rate of greater than or equal to 60 beats per minute)[22].

Ventricular tachycardia was defined as three or more beats of ventricular origin in succession at a rate greater than 100 beats/minute; ventricular fibrillation defined as irregular undulations of varying contour and amplitude on the ECG, with absent distinct QRS and T waves and hemodynamic compromise requiring direct-current defibrillation.

Congestive heart failure (CHF) are defined by the symptoms: exertional dyspnea, orthopnea, shortness of breath, labored breathing, fatigue at either rest or with exertion; and signs: rales greater than one-third of the lung fields, S_3 _gallop on auscultation, or pulmonary congestion on X-ray film.

### Statistical analysis

Baseline characteristics are expressed as mean ± SD or percentages. The ANOVA (analysis of variance) for continuous variables was used to compare baseline data among the groups. Chi-square test was used to compare percentages among the groups. To evaluate the independent relationship between hemoglobin and MACEs at 30 days, multivariable logistic regression (logistic modeling with categorical predictors) was used. Hemoglobin was coded as a multicategory predictor in 10 g/L increments, and the hemoglobin category that had the lowest event rate and normal hemoglobin level was used as the reference group. This approach yielded the following covariates: age; gender; past history: hypertension, diabetes, hyperlipidemia, coronary heart disease, smoking history; the type of current AMI: STE or NSTE; heart rate, diastolic pressure, pulse pressure, LVEF, albumin, creatinine, CK-MB, total cholesterol (TC), triglyceride (TG), low density lipoprotein cholesterol (LDL-C), high density lipoprotein cholesterol (HDL-C); index medications: aspirin, β-blocker, calcium channel blockers (CCB), nitrates, angiotensin converting enzyme inhibitory (ACEI), angiotensin receptor blocker (ARB), statin, thrombolysis, anticoagulation and index PCI for AMI patients. Zero-order correlation analysis (no taking account of covariates) and partial correlation analysis (taking account of covariates) were used to analysis the relation between hemoglobin concentration and related factors including age, heart rate, blood pressure, pulse pressure, LVEF, albumin, creatinine, CK-MB, TC, TG, HDL-C and LDL-C. A value of P < 0.05 was considered significant. Statistical analysis were performed with SAS for Windows version 9.1(SAS Institute, Cary, N.C. USA) and SPSS for Windows version 13(SPSS Inc., Chicago, IL. USA).

## Results

### The differences in baseline characteristics among the groups

Patients baseline characteristics, included age, gender, heart rate, blood pressure, LVEF and laboratory data, among eight groups are outlined in Table 1. The differences in age, gender, diastolic pressure, pulse pressure, LVEF, albumin, creatinine, TC, TG, and LDL-C among the eight groups were significant.

**Table 1:** Baseline Characteristics in AMI Patients Stratified by Baseline Hemoglobin

Variable	<100 g/L (n = 86)	101-110 g/L (n = 74)	111-120 g/L (n = 143)	121-130 g/L (n = 222)	131-140 g/L (n = 278)	141-150 g/L (n = 296)	151-160 g/L (n = 173)	>160 g/L (n = 122)	*p *value
Age (year)	74.0 ± 10.2	71.8 ± 11.7	72.2 ± 11.7	67.5 ± 12.2	63.6 ± 12.0	57.9 ± 12.9	52.7 ± 12.3	53.0 ± 11.8	0.000
Gender(female/male)	36/50	29/45	49/94	61/161	43/235	26/270	7/166	5/117	0.000
Hb(g/L)	86.3 ± 12.1	106.3 ± 2.6	115.9 ± 2.8	125.6 ± 2.8	135.6 ± 2.9	145.4 ± 3.0	155.3 ± 2.9	167.7 ± 6.3	0.000
Heart rate(beats/min)	82.3 ± 20.4	81.4 ± 19.3	79.3 ± 16.3	78.4 ± 17.3	77.5 ± 15.2	75.7 ± 13.4	76.7 ± 13.6	78.5 ± 17.8	0.011
Systolic pressure(mmHg)	127.7 ± 25.7	120.9 ± 22.5	126.2 ± 22.9	123.1 ± 21.2	123.9 ± 20.4	123.2 ± 18.9	125.3 ± 18.1	126.4 ± 21.6	0.281
Diastolic pressure(mmHg)	72.7 ± 15.4	69.2 ± 13.0	71.4 ± 14.8	72.1 ± 12.4	73.5 ± 11.6	73.6 ± 12.7	77.6 ± 11.5	77.8 ± 14.9	0.000
Pulse pressure(mmHg)	55.0 ± 17.3	51.7 ± 18.0	54.8 ± 16.4	51.1 ± 16.1	50.4 ± 17.0	49.6 ± 13.9	47.8 ± 13.1	48.7 ± 14.8	0.000
LVEF(%)	48.9 ± 12.0	47.0 ± 12.0	47.5 ± 11.1	49.0 ± 11.1	51.3 ± 11.3	52.2 ± 9.7	52.6 ± 9.1	53.0 ± 10.4	0.000
Albumin (g/L)	32.6 ± 4.6	33.3 ± 4.6	35.7 ± 4.5	36.9 ± 3.9	37.7 ± 4.1	39.7 ± 4.1	41.2 ± 4.0	41.5 ± 4.3	0.000
Creatinine (μmol/L)	139.9 ± 30.8	101.0 ± 71.9	89.4 ± 56.4	81.1 ± 45.7	77.6 ± 26.7	80.0 ± 51.9	71.9 ± 25.0	81.3 ± 48.0	0.000
CK-MB(u/L)	115.9 ± 194.1	113.8 ± 164.5	132.3 ± 171.3	133.5 ± 200.2	148.9 ± 190.6	143.3 ± 216.0	166.8 ± 211.1	173.1 ± 193.1	0.499
TC(mmol/L)	4.5 ± 1.3	4.4 ± 1.1	4.5 ± 1.3	4.5 ± 1.1	4.6 ± 1.1	4.7 ± 1.1	4.8 ± 1.1	4.8 ± 1.3	0.026
TG(mmol/L)	1.6 ± 1.4	1.5 ± 1.0	1.3 ± 0.6	1.5 ± 1.2	1.7 ± 1.0	1.8 ± 1.0	2.0 ± 1.2	2.1 ± 1.3	0.000
HDL-C(mmol/L)	1.2 ± 0.4	1.2 ± 0.3	1.1 ± 0.3	1.2 ± 0.3	1.2 ± 0.6	1.2 ± 0.3	1.1 ± 0.3	1.2 ± 0.3	0.871
LDL-C(mmol/L)	2.6 ± 1.1	2.2 ± 0.8	2.6 ± 1.1	2.6 ± 0.9	2.7 ± 0.9	2.7 ± 0.9	2.8 ± 0.8	2.8 ± 1.1	0.013

TC: total cholesterol; TG: triglyceride; HDL-C: high density lipoprotein cholesterol; LDL-C: low density lipoprotein cholesterol. The data except those in gender item are expressed as mean ± SD.

The baseline characteristics, included past history, smoking history, family history of CHD, myocardial infarction and medication among eight groups are outlined in Table 2. The differences in past history of diabetes mellitus, hyperlipidemia and CHD, smoking history, family history of CHD, non-Q wave myocardial infarction, aspirin, β-blocker, statin, thrombolysis and PCI among the eight groups were significant.

**Table 2:** Risk factor, past history, the location of AMI and medication in different group

Variable	<100 g/L (n = 86)	101-110 g/L (n = 74)	111-120 g/L (n = 143)	121-130 g/L (n = 222)	131-140 g/L (n = 278)	141-150 g/L (n = 296)	151-160 g/L (n = 173)	>160 g/L (n = 122)	*p *value
Past history									
Hypertension(%)	61 (70.9)	46 (62.2)	79 (55.2)	124 (55.9)	143 (51.4)	159 (53.7)	88 (50.9)	65 (53.3)	0.064
Diabetes mellitus(%)	40 (46.5)	19 (25.7)	48 (33.6)	64 (28.8)	58 (20.9)	52 (17.6)	32 (18.5)	18 (14.8)	0.000
Hyperlipidemia(%)	27 (31.8)	29 (39.1)	39 (27.4)	76 (34.2)	111 (39.9)	142 (47.9)	99 (57.0)	74 (60.7)	0.000
CAD(%)	36 (41.9)	28 (37.8)	50 (35.0)	74 (33.3)	69 (24.8)	81 (27.4)	34 (19.7)	30 (24.6)	0.001
Smoking history(%)	16 (18.6)	20 (27.0)	45 (31.5)	71 (32.0)	144 (51.8)	138 (46.6)	97 (56.1)	71 (58.2)	0.000
Family history of CHD(%)	13 (15.1)	16 (21.6)	26 (18.2)	54 (24.3)	78 (28.1)	97 (32.8)	55 (31.8)	38 (31.1)	0.003
Location of AMI									
Anterior wall(%)	21 (24.4)	25 (33.8)	45 (31.5)	75 (33.8)	98 (35.3)	123 (41.6)	69 (39.9)	55 (45.1)	0.025
Inferior wall(%)	18 (20.9)	26 (35.1)	46 (32.2)	57 (25.7)	98 (35.3)	100 (33.8)	53 (30.6)	36 (29.5)	0.129
Other wall(%)	9 (10.5)	9 (12.2)	14 (9.8)	26 (11.7)	35 (19.9)	43 (14.5)	21 (12.1)	24 (19.7)	0.356
Non-Q wave MI(%)	38 (44.2)	22 (29.7)	39 (27.3)	71 (32.0)	69 (24.8)	53 (17.9)	31 (17.9)	25 (20.5)	0.000
Indexed medication									
Aspirin(%)	59 (68.6)	58 (78.4)	128 (89.5)	203 (91.4)	250 (89.9)	280 (94.6)	164 (94.8)	113 (92.6)	0.000
β-blocker(%)	46 (53.5)	34 (45.9)	69 (48.3)	125 (56.3)	181 (65.1)	187 (63.2)	104 (60.1)	72 (59.0)	0.005
CCB(%)	22 (25.6)	17 (23.0)	46 (47.4)	46 (20.7)	60 (21.6)	71 (24.0)	38 (22.0)	27 (22.1)	0.322
Nitrates(%)	62 (72.1)	52 (70.3)	116 (81.1)	162 (73.0)	207 (74.5)	207 (69.9)	115 (66.5)	86 (70.5)	0.170
ACEI(%)	44 (51.2)	39 (52.7)	74 (51.7)	111 (50.0)	156 (56.1)	179 (60.5)	110 (63.6)	69 (56.6)	0.104
ARB(%)	6 (7.0)	5 (6.8)	8 (5.6)	11 (5.0)	13 (4.7)	19 (6.4)	9 (5.2)	7 (5.7)	0.981
Statin(%)	25 (29.1)	23 (31.1)	57 (39.9)	98 (44.1)	128 (46.0)	160 (54.1)	92 (53.2)	68 (55.7)	0.000
Thrombolysis(%)	4 (4.7)	6 (8.1)	10 (7.0)	27 (12.2)	40 (14.4)	56 (18.9)	27 (15.6)	20 (16.4)	0.002
Anticoagulation(%)	47 (54.7)	42 (56.8)	84 (58.7)	133 (59.9)	179 (64.4)	185 (62.5)	113 (65.3)	87 (71.3)	0.198
PCI(%)	20 (23.3)	33 (44.6)	48 (33.6)	120 (54.1)	178 (64.0)	231 (78.0)	145 (83.8)	93 (76.2)	0.000

CAD: coronary artery disease; CHD: coronary heart disease; CCB: calcium channel blockers; ACEI: ACE inhibitor; ARB: angiotensin receptor blocker; PCI: percutaneous coronary intervention. The numbers but those in the second line in parentheses are expressed in percentage.

Among the groups, those with lower baseline hemoglobin concentrations were more likely to be older, past history of CAD, non-Q wave myocardial infarction and diabetes; and less likely to be a smoker and hyperlipidemia, and to have family history of CHD; and had lower LVEF, lower albumin, lower total cholesterol, lower triglyceride, lower diastolic pressure, higher pulse pressure and higher creatinine.

### The association between hemoglobin concentration and clinical outcomes

#### Unadjusted clinical outcomes rates

The cardiac death and complications within 30 days after admission in the eight groups are shown in Table 3. The group with lower hemoglobin concentration on admission had higher cardiac death, and more likely suffered from cardiogenic shock, congestive heart failure and postinfarction angina, than the groups with higher hemoglobin concentration on admission.

**Table 3:** MACEs through 30 days

Variable	<100 g/L (n = 86)	101-110 g/L (n = 74)	111-120 g/L (n = 143)	121-130 g/L (n = 222)	131-140 g/L (n = 278)	141-150 g/L (n = 296)	151-160 g/L (n = 173)	>160 g/L (n = 122)	p value
Cardiogenic shock(%)	9(10.5)	7(9.5)	10(7.0)	16(7.2)	16(5.8)	8(2.7)	5(2.9)	6(4.9)	0.0367
Heart failure(%)	31(36.0)	21(28.4)	24(16.8)	42(18.9)	34(12.2)	24(8.1)	15(8.7)	9(7.4)	<0.0001
Postinfarction angina(%)	3(3.5)	8(10.8)	7(4.9)	9(4.1)	7(2.5)	5(1.7)	2(1.2)	5(4.1)	0.0050
VT/VF(%)	5(5.8)	5(6.8)	10(7.0)	12(5.4)	14(5.0)	16(5.4)	12(6.9)	6(4.9)	0.9813
Cardiac death(%)	17(19.8)	13(17.6)	21(14.7)	21(9.5)	21(7.6)	9(3.0)	6(3.5)	10(8.2)	<0.0001
MACEs(%)	41(47.7)	29(39.2)	46(32.2)	61(27.5)	56(20.1)	44(14.9)	29(16.8)	24(36.1)	<0.0001

The numbers but those in the second line in parentheses are expressed in percentage.

**Figure 1:**
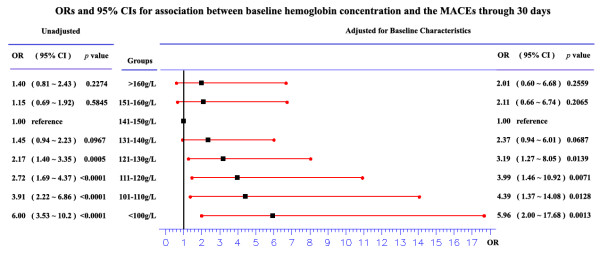
**ORs and 95% CIs for association between baseline hemoglobin concentration and the MACEs through 30 days in patients with AMI **. Adjusted for gender; age; past history: hypertension, diabetes, hyperlipidemia, CAD, smoking history; the type of current AMI: STE or NSTE; heart rate; diastolic pressure; pulse pressure; LVEF; albumin; creatinine; CK-MB; TC; TG; LDL-C; HDL-C; index medications: aspirin, β-blocker, CCB, nitrates, ACE inhibitor, ARB, statin, thrombolysis, anticoagulation and PCI.

The likelihood of cardiogenic shock, heart failure, postinfarction angina, and cardiac death was significantly related to baseline hemoglobin (Table 3, *P *= 0.0367, <0.0001, 0.005, and <0.0001 for cardiogenic shock, heart failure, postinfarction angina, and cardiac death respectively), with the patients at either end of the hemoglobin spectrum being more likely to have adverse clinical outcome: cardiac death and cardiogenic shock. Figure 1 shows the unadjusted OR and 95% CI for 30-day MACEs in patients with AMI categorized by 10 g/L hemoglobin increments.

#### Adjusted clinical outcomes rates

Considering imbalances in important baseline characteristics, multivariable logistic regression was used to evaluate the independent relationship between hemoglobin and MACEs after adjustment for a wide range of covariates, including age; gender; past history: hypertension, diabetes, hyperlipidemia, CAD, smoking history; the type of current AMI: STE or NSTE; heart rate; diastolic pressure; pulse pressure; LVEF; albumin; creatinine; CK-MB; TC; TG; LDL-C; HDL-C; index medications: aspirin, β-blocker, CCB, nitrates, ACE inhibitor, ARB, statin, thrombolysis, anticoagulation and PCI. Figure 1 shows the adjusted OR and 95% CI for 30-day MACEs in patients with AMI categorized by 10 g/L hemoglobin increments. With the patients with hemoglobin values of 141 to 150 g/L as the reference, the MACEs increased as hemoglobin levels fell below 140 g/L, with an adjusted OR of 5.96(95% CI 2.00 to 17.68, p = 0.0013), 4.39(1.37 to 14.08, p = 0.0128), 3.99(1.46 to 10.92, p = 0.0071), 3.19(1.27 to 8.05, p = 0.0139), 2.37(0.94 to 6.01, p = 0.0687) in the patients with haemoglobin concentrations <100 g/L, 101-110 g/L, 111-120 g/L, 121-130 g/L, 131-140 g/L, respectively; the MACEs had a increased tendency as hemoglobin levels were above 150 g/L, with an adjusted OR of 2.11(0.66 to 6.74, p = 0.2065), 2.01(0.60 to 6.68, p = 0.2559) in the patients with haemoglobin concentrations 151-160 g/L and >160 g/L respectively, as shown in Figure 1.

#### The factors related to hemoglobin

Zero-order correlation analysis showed that age, diastolic pressure, pulse pressure, TG, albumin, creatinine, and LVEF were significantly associated with hemoglobin concentration; partial correlation analysis showed that age, albumin and creatinine were significantly associated with hemoglobin concentration after controlling related factors, with partial correlation probability of <0.001, 0.001 and <0.001 respectively, as shown in Table 4. The controlling factors for the partial correlation included following covariates: age, heart rate, systolic and diastolic pressure, pulse pressure, albumin, creatinine, LVEF, CK-MB, TC, TG, HDL-C and LDL-C.

**Table 4:** Zero order and partial correlation analysis of factors related to hemoglobin

Variable	Mean ± SD	Zero-order correlation coefficient	Zero-order correlation probability	Partial correlation coefficient	Partial correlation probability
Age	62.2 ± 13 years	-0.530	0.000	-0.368	0.000
Diastolic pressure	71.3 ± 13.2 mmHg	0.174	0.007	0.000	1.000
Pulse pressure	53.8 ± 14.8 mmHg	-0.177	0.007	0.000	1.000
Albumin	37.7 ± 4.3 g/L	0.447	0.000	0.226	0.001
Creatinine	87.4 ± 59.2 mmol/L	-0.339	0.000	-0.288	0.000
TG	1.5 ± 0.9 mmol/L	0.210	0.001	0.017	0.798
LVEF	50.7 ± 11.1%	0.148	0.023	0.082	0.226

## Discussions

Haemoglobin, as a main oxygen carrier, plays an important role in supplying oxygen to tissues. When haemoglobin decreases, body may increase cardiac output to maintain the normal metabolic demands of tissues, which increases work load of heart, and result in myocardial damage [4,5].

### The association between haemoglobin concentration and clinical outcomes

The relationship between hemoglobin concentrations and cardiovascular outcomes has been reported in a broad cohort of patients with ACS [15,17,23,24]. But the association between low hemoglobin concentrations and adverse cardiovascular outcomes has not been settled.

Al Falluji et al [15] examined a database of discharge abstract information in patients admitted with myocardial infarction included 30,341 patients hospitalized in 1986 and (prethrombolytic era, n = 15,584) and 1996 (thrombolytic era, n = 14,757), anemia in this study were not found to have a higher mortality. In contrast, in a large database study of elderly patients with AMI, a powerful, albeit unadjusted, relationship between hematocrit on admission and all-cause 30-day mortality was found, there was a dose-response effect, with progressively lower survival rates with more profound degrees of anemia[12]. Similar results was also reported in the research by Sabatine et al[17], which included 25419 patients with ST-segment elevation myocardial infarction (STEMI) and 14 503 patients with NSTE from 16 Thrombolysis In Myocardial Infarction trials, and a reverse J-shaped relationship between hemoglobin concentrations and clinical outcomes was found: in patients with STEMI, the cardiovascular mortality increased as hemoglobin concentrations fell below 14 g/dL and hemoglobin values rose above 17 g/dL, compared with hemoglobin concentrations between 14 and 15 g/dL; in patients with NSTE ACS, the cardiovascular death, myocardial infarction, or recurrent ischemia increased as the hemoglobin fell below 11 g/dL and hemoglobin values rose above 16 g/dL, compared with those with hemoglobin 15 to 16 g/dL.

But in Chinese AMI, the relationship between hemoglobin concentrations and cardiovascular outcomes has not been reported up till now. In this research of Chinese AMI, from single medical center, a reverse J-shaped (Figure 1) relationship between hemoglobin concentrations and clinical outcomes was also found, with patients at either end of the hemoglobin spectrum being more likely to have adverse clinical outcomes, significantly for those with haemoglobin concentrations <140 g/L, insignificantly but tendentiously for with haemoglobin concentrations >150 g/L. Compared with patients with haemoglobin concentrations 141-150 g/L, those with haemoglobin concentrations <140 g/L had more cardiac death, cardiogenic shock and heart failure, and those with haemoglobin concentrations >150 g/L also had more cardiac death, cardiogenic shock and postinfarction angina. Through Sabatine and his colleagues[17] have reported a similar results, this report adjusted some important risk factors for MACEs, such as CK-MB and LVEF, which were not adjusted in Sabatine and his colleagues' research. Otherwise, our research had different clinical outcomes, which included cardiogenic shock and heart failure.

The worse outcomes observed in patients with AMI with either end of the hemoglobin spectrum might be explained by theory that anemia could decrease oxygen delivery to tissues, therefore attenuate the ability of collateral flow from nearby patent vessels to limit the extent of myocardial necrosis and peri-infarct ischemia, meanwhile anemia increase myocardial oxygen demand through necessitating a higher work load; and that higher haemoglobin concentration would increase blood viscosity, which decrease oxygen delivery to tissues [25].

### The factors related to haemoglobin concentration

Anemia is common in elder people, about 12.5% of patients aged 71 years or older had anemia [26]. Anemia accounted for 24% of geriatric hospitalized population in some reports [2]. A positive relationship between haemoglobin concentration and serum albumin concentration was reported in the elderly [27]. A direct relationship between haemoglobin concentration and serum albumin level was found in hemodialysis patients [28,29]. Age was also found to be significantly associated with hemoglobin concentrations (P < 0.001) [29]. But the association between age and haemoglobin concentration has not been reported in AMI patients.

In this paper, the factors related to haemoglobin concentrations were analysed. Age, diastolic pressure, TG, albumin and LVEF were found to be significantly associated with hemoglobin concentration (zero-order correlation probability < 0.01); After careful controlling these relevant factors, age, creatinine, and albumin were significantly associated with hemoglobin concentration (partial correlation probability < 0.01). The older, those with higher creatinine level and those with lower albumin level were more likely to have lower hemoglobin concentrations. Hemoglobin concentration falls with age and creatinine increase, and rises with albumin increase.

There are several possible explanations for these findings. First, elderly people might easily suffer from anemia because hematogenous function declines with aging [30-32]. Second, albumin is a marker of nutrition. Low albumin level means protein-calorie malnutrition to some extent [33,34]. Shortened red blood cells survival, decreased erythropoietin secretion by kidney, and concurrent deficiencies of iron, pyridoxine and folate have been reported to contribute to anemia [34].

Although the association between hemoglobin concentrations and adverse cardiovascular events was demonstrated in this and other researches [17,18,35], the beneficial effect of transfusion on clinical outcomes remain to be settled.

The studies of transfusion effect on clinical outcomes of anemia have yielded conflicting results. In elderly patients with AMI, transfusion appeared to be beneficial if the hematocrit was < 33% [12]. In contrast, Transfusion was reported to have adverse impact on the prognosis of acute coronary syndromes in some nonrandomized trails. Transfusion in anemic patients admitted with acute coronary syndrome led to a significant increase in 30-day recurrent MI or death [19,20], especially for NSTE ACS [17]. Therefore, transfusion effect on clinical outcomes of anemia remains to be further investigated.

### The limitation of this study

As this is a retrospective study, some potential limitations of this study should be considered. The present study was from single large Chinese medical center, which might limit generalizability of our findings. Management strategy was not included in variables. There may be some differences among groups in management strategy, as was reported in some studies [17]. Differences in treatment among the groups might have the potential to confound our analyses. The cause of anemia in patients in the present study was not known, the different causes might have residual confounding. Because the active bleeding was not included in this study, the anemia exactly means chronic anemia. Although we cannot rule out the possibility that we were unable to adjust for this known and other unknown confounders, given the breadth of covariates adjusted for in the present analyses, the impact is likely to be small.

Since this data was from single large center, which avoid the bias from different centers, unity of data was guaranteed.

## Conclusions

Anemia and too high hemoglobin concentrations might lead to adverse clinical outcome, and age, albumin and creatinine are significantly associated with anemia. So we should pay much attention to the AMI with anemia and the elderly to improve the prognosis.

## List of abbreviations

AMI: acute myocardial infarction; Hb: haemoglobin; PCI: percutaneous coronary intervention; ACS: acute coronary syndrome; CHD: coronary heart disease; LVEF: left ventricular ejection fraction; MACEs: major cardiovascular events; ECG: Electrocardiogram; CHF: congestive heart failure; ANOVA: analysis of variance; CK-MB: creatine kinase-MB; TC: total cholesterol; TG: triglyceride; LDL-C: low density lipoprotein cholesterol; HDL-C: high density lipoprotein cholesterol; CCB: calcium channel blockers; ACEI: angiotensin converting enzyme inhibitory; ARB: angiotensin receptor blocker.

## Competing interests

The authors declare that they have no competing interests.

## Authors' contributions

QZ designed the study, analysed the data, interpreted the results and wrote the manuscript. YS was involved in conception and data collection, and design of the study. YF was involved in data collection, and interpretation of results. All authors read and approved the final manuscript.

## References

[B1] Chesebro JH Knatterud G Roberts R Borer J Cohen LS Dalen J Dodge HT Francis CK Hillis D Ludbrook P Thrombolysis in Myocardial Infarction (TIMI) Trial, Phase I: A comparison between intravenous tissue plasminogen activator and intravenous streptokinase. Clinical findings through hospital discharge Circulation 1987 76 142 54 3109764 10.1161/01.cir.76.1.142

[B2] Joosten E Pelemans W Hiele M Noyen J Verhaeghe R Boogaerts MA Prevalence and causes of anaemia in a geriatric hospitalized population Gerontology 1992 38 111 7 10.1159/000213315 1612458

[B3] Most AS Ruocco NA Jr Gewirtz H Effect of a reduction in blood viscosity on maximal myocardial oxygen delivery distal to a moderate coronary stenosis Circulation 1986 74 1085 92 3769166 10.1161/01.cir.74.5.1085

[B4] Levy PS Quigley RL Gould SA Acute dilutional anemia and critical left anterior descending coronary artery stenosis impairs end organ oxygen delivery J Trauma 1996 41 416 23 10.1097/00005373-199609000-00006 8810957

[B5] Yoshikawa H Powell WJ Jr Bland JH Lowenstein E Effect of acute anemia on experimental myocardial ischemia Am J Cardiol 1973 32 670 8 10.1016/S0002-9149(73)80061-X 4744695

[B6] Levy PS Kim SJ Eckel PK Chavez R Ismail EF Gould SA Ramez SM Crystal GJ Limit to cardiac compensation during acute isovolemic hemodilution: influence of coronary stenosis Am J Physiol 1993 265 H340 9 8342651 10.1152/ajpheart.1993.265.1.H340

[B7] Hu H Xenocostas A Chin-Yee I Lu X Feng Q Effects of anemia and blood transfusion in acute myocardial infarction in rats Transfusion 2010 50 243 251 10.1111/j.1537-2995.2009.02385.x 19778337

[B8] Sarnak MJ Tighiouart H Manjunath G MacLeod B Griffith J Salem D Levey AS Anemia as a risk factor for cardiovascular disease in The Atherosclerosis Risk in Communities (ARIC) study J Am Coll Cardiol 2002 40 27 33 10.1016/S0735-1097(02)01938-1 12103252

[B9] Al-Ahmad A Rand WM Manjunath G Konstam MA Salem DN Levey AS Sarnak MJ Reduced kidney function and anemia as risk factors for mortality in patients with left ventricular dysfunction J Am Coll Cardiol 2001 38 955 62 10.1016/S0735-1097(01)01470-X 11583864

[B10] Ezekowitz JA McAlister FA Armstrong PW Anemia is common in heart failure and is associated with poor outcomes: insights from a cohort of 12 065 patients with new-onset heart failure Circulation 2003 107 223 5 10.1161/01.CIR.0000052622.51963.FC 12538418

[B11] McKechnie RS Smith D Montoye C Kline-Rogers E O'Donnell MJ DeFranco AC Meengs WL McNamara R McGinnity JG Patel K Share D Riba A Khanal S Moscucci M Prognostic implication of anemia on in-hospital outcomes after percutaneous coronary intervention Circulation 2004 110 271 7 10.1161/01.CIR.0000134964.01697.C7 15226214

[B12] Wu WC Rathore SS Wang Y Radford MJ Krumholz HM Blood transfusion in elderly patients with acute myocardial infarction N Engl J Med 2001 345 1230 6 10.1056/NEJMoa010615 11680442

[B13] Lee PC Kini AS Ahsan C Fisher E Sharma SK Anemia is an independent predictor of mortality after percutaneous coronary intervention J Am Coll Cardiol 2004 44 541 6 10.1016/j.jacc.2004.04.047 15358017

[B14] Nikolsky E Aymong ED Halkin A Grines CL Cox DA Garcia E Mehran R Tcheng JE Griffin JJ Guagliumi G Stuckey T Turco M Cohen DA Negoita M Lansky AJ Stone GW Impact of anemia in patients with acute myocardial infarction undergoing primary percutaneous coronary intervention: analysis from the Controlled Abciximab and Device Investigation to Lower Late Angioplasty Complications (CADILLAC) Trial J Am Coll Cardiol 2004 44 547 53 10.1016/j.jacc.2004.03.080 15358018

[B15] Al FN Lawrence-Nelson J Kostis JB Lacy CR Ranjan R Wilson AC Effect of anemia on 1-year mortality in patients with acute myocardial infarction Am Heart J 2002 144 636 41 12360159 10.1067/mhj.2002.124351

[B16] Nabais S Gaspar A Costa J Azevedo P Rocha S Torres M Pereira MA Correia A Prognostic impact of hemoglobin drop during hospital stay in patients with acute coronary syndromes Rev Port Cardiol 2009 28 383 95 19634496

[B17] Sabatine MS Morrow DA Giugliano RP Burton PB Murphy SA McCabe CH Gibson CM Braunwald E Association of hemoglobin levels with clinical outcomes in acute coronary syndromes Circulation 2005 111 2042 9 10.1161/01.CIR.0000162477.70955.5F 15824203

[B18] Aronson D Suleiman M Agmon Y Suleiman A Blich M Kapeliovich M Beyar R Markiewicz W Hammerman H Changes in haemoglobin levels during hospital course and long-term outcome after acute myocardial infarction Eur Heart J 2007 28 1289 96 10.1093/eurheartj/ehm013 17363447

[B19] Rao SV Jollis JG Harrington RA Granger CB Newby LK Armstrong PW Moliterno DJ Lindblad L Pieper K Topol EJ Stamler JS Califf RM Relationship of blood transfusion and clinical outcomes in patients with acute coronary syndromes JAMA 2004 292 1555 62 10.1001/jama.292.13.1555 15467057

[B20] Singla I Zahid M Good CB Macioce A Sonel AF Impact of blood transfusions in patients presenting with anemia and suspected acute coronary syndrome Am J Cardiol 2007 99 1119 21 10.1016/j.amjcard.2006.11.056 17437739

[B21] Alpert JS Thygesen K Antman E Bassand JP Myocardial infarction redefined--a consensus document of The Joint European Society of Cardiology/American College of Cardiology Committee for the redefinition of myocardial infarction J Am Coll Cardiol 2000 36 959 69 10.1016/S0735-1097(00)00804-4 10987628

[B22] Cannon CP Battler A Brindis RG Cox JL Ellis SG Every NR Flaherty JT Harrington RA Krumholz HM Simoons ML Van De Werf FJ Weintraub WS Mitchell KR Morrisson SL Brindis RG Anderson HV Cannom DS Chitwood WR Cigarroa JE Collins-Nakai RL Ellis SG Gibbons RJ Grover FL Heidenreich PA Khandheria BK Knoebel SB Krumholz HL Malenka DJ Mark DB Mckay CR Passamani ER Radford MJ Riner RN Schwartz JB Shaw RE Shemin RJ Van Fossen DB Verrier ED Watkins MW Phoubandith DR Furnelli T American College of Cardiology key data elements and definitions for measuring the clinical management and outcomes of patients with acute coronary syndromes. A report of the American College of Cardiology Task Force on Clinical Data Standards (Acute Coronary Syndromes Writing Committee) J Am Coll Cardiol 2001 38 2114 30 10.1016/S0735-1097(01)01702-8 11738323

[B23] Archbold RA Balami D Al-Hajiri A Suliman A Liew R Cooper J Ranjadayalan K Knight CJ Deaner A Timmis AD Hemoglobin concentration is an independent determinant of heart failure in acute coronary syndromes: cohort analysis of 2310 patients Am Heart J 2006 152 1091 5 10.1016/j.ahj.2006.07.020 17161058

[B24] Cavusoglu E Chopra V Gupta A Clark LT Eng C Marmur JD Usefulness of anemia in men as an independent predictor of two-year cardiovascular outcome in patients presenting with acute coronary syndrome Am J Cardiol 2006 98 580 4 10.1016/j.amjcard.2006.03.031 16923440

[B25] Gustavsson CG Persson S Thorvinger BO Hedner P Association between pre-PTCA blood haemoglobin concentration and the risk of symptomatic restenosis after successful PTCA of primary coronary stenoses J Cardiovasc Risk 1997 4 37 40 9215519 10.1177/174182679700400107

[B26] Penninx BW Pahor M Woodman RC Guralnik JM Anemia in old age is associated with increased mortality and hospitalization J Gerontol A Biol Sci Med Sci 2006 61 474 9 16720744 10.1093/gerona/61.5.474

[B27] Eliana F Soejono CH Widjanarko A Albar Z Bachtiar A Iron deposit state and risk factors for anemia in the elderly Acta Med Indones 2005 37 118 25 16138416

[B28] Rocco MV Bedinger MR Milam R Greer JW McClellan WM Frankenfield DL Duration of dialysis and its relationship to dialysis adequacy, anemia management, and serum albumin level Am J Kidney Dis 2001 38 813 23 10.1053/ajkd.2001.27701 11576885

[B29] Madore F Lowrie EG Brugnara C Lew NL Lazarus JM Bridges K Owen WF Anemia in hemodialysis patients: variables affecting this outcome predictor J Am Soc Nephrol 1997 8 1921 9 9402095 10.1681/ASN.V8121921

[B30] Kario K Matsuo T Kodama K Nakao K Asada R Reduced erythropoietin secretion in senile anemia Am J Hematol 1992 41 252 7 10.1002/ajh.2830410406 1288287

[B31] Carpenter MA Kendall RG O'Brien AE Chapman C Sebastian JP Belfield PW Norfolk DR Reduced erythropoietin response to anaemia in elderly patients with normocytic anaemia Eur J Haematol 1992 49 119 21 1446724 10.1111/j.1600-0609.1992.tb00914.x

[B32] Lee MA Segal GM Bagby GC The hematopoietic microenvironment in the elderly: defects in IL-1-induced CSF expression in vitro Exp Hematol 1989 17 952 6 2476327

[B33] Walsh JR Cassel CK, Cohen HJ, Larson EB, et al Hematologic problem Geriatric Medicine 1997 3 New York, USA: Springer-Verlag 627 636

[B34] Lipschitz DA Protein calorie malnutrition in the hospitalized elderly Prim Care 1982 9 531 43 6815677

[B35] Rao SV Eikelboom JA Granger CB Harrington RA Califf RM Bassand JP Bleeding and blood transfusion issues in patients with non-ST-segment elevation acute coronary syndromes Eur Heart J 2007 28 1193 204 10.1093/eurheartj/ehm019 17456480

